# Opsonized antigen activates Vδ2+ T cells via CD16/FCγRIIIa in individuals with chronic malaria exposure

**DOI:** 10.1371/journal.ppat.1008997

**Published:** 2020-10-21

**Authors:** Lila A. Farrington, Perri C. Callaway, Hilary M. Vance, Kayla Baskevitch, Emma Lutz, Lakshmi Warrier, Tara I. McIntyre, Rachel Budker, Prasanna Jagannathan, Felistas Nankya, Kenneth Musinguzi, Mayimuna Nalubega, Ester Sikyomu, Kate Naluwu, Emmanuel Arinaitwe, Grant Dorsey, Moses R. Kamya, Margaret E. Feeney

**Affiliations:** 1 Department of Medicine, University of California San Francisco, San Francisco, California, United States of America; 2 Infectious Disease and Immunity Graduate Group, University of California Berkeley, California, United States of America; 3 Department of Medicine, Stanford University, Stanford, California, United States of America; 4 Infectious Diseases Research Collaboration, Kampala, Uganda; 5 College of Health Sciences, Makerere University, Kampala, Uganda; 6 Department of Pediatrics, University of California San Francisco, San Francisco, California, United States of America; UNITED STATES

## Abstract

Vγ9Vδ2 T cells rapidly respond to phosphoantigens produced by *Plasmodium falciparum* in an innate-like manner, without prior antigen exposure or processing. Vδ2 T cells have been shown to inhibit parasite replication *in vitro* and are associated with protection from *P*. *falciparum* parasitemia *in vivo*. Although a marked expansion of Vδ2 T cells is seen after acute malaria infection in naïve individuals, repeated malaria causes Vδ2 T cells to decline both in frequency and in malaria-responsiveness, and to exhibit numerous transcriptional and phenotypic changes, including upregulation of the Fc receptor CD16. Here we investigate the functional role of CD16 on Vδ2 T cells in the immune response to malaria. We show that CD16+ Vδ2 T cells possess more cytolytic potential than their CD16- counterparts, and bear many of the hallmarks of mature NK cells, including KIR expression. Furthermore, we demonstrate that Vδ2 T cells from heavily malaria-exposed individuals are able to respond to opsonized *P*.*falciparum*-infected red blood cells through CD16, representing a second, distinct pathway by which Vδ2 T cells may contribute to anti-parasite effector functions. This response was independent of TCR engagement, as demonstrated by blockade of the phosphoantigen presenting molecule Butyrophilin 3A1. Together these results indicate that Vδ2 T cells in heavily malaria-exposed individuals retain the capacity for antimalarial effector function, and demonstrate their activation by opsonized parasite antigen. This represents a new role both for Vδ2 T cells and for opsonizing antibodies in parasite clearance, emphasizing cooperation between the cellular and humoral arms of the immune system.

## Author summary

T cells that express the Vδ2 and Vγ9 TCR chains (Vδ2 T cells) have been shown to play an important role in controlling parasitemia during *P*.*falciparum* infection. A better understanding of how these cells interact with malaria parasites to control infection is necessary. We have previously shown that after multiple *P*. *falciparum* infections, Vδ2 T cells decrease in frequency, become less responsive to TCR stimulation, and upregulate the Fc receptor CD16. Here we investigate whether Vδ2 T cells from chronically malaria-exposed individuals can be activated directly through CD16 to release proinflammatory cytokines and degranulate. We show that in these individuals, TCR is downregulated on CD16+ Vδ2 T cells, and that these cells are more likely to express a variety of cytotoxic effector molecules. Importantly, we show that these CD16+ Vδ2 T cells can be activated directly through CD16, independent of TCR, by antibody bound to parasite antigen. These results are notable because they indicate many Vδ2 T cells from chronically-exposed individuals may not be exhausted but instead favor an alternative activation pathway, one that cooperates with a mature anti-malarial antibody response.

## Introduction

γδ T cells are believed to play an important role in the immune response to malaria. These unconventional lymphocytes comprise up to 5% of peripheral blood T cells and exhibit features of both adaptive and innate immune cells. γδ T cells expressing the Vδ2 and Vγ9 TCR chains are intrinsically reactive to malaria due to their activation by low-molecular weight phosphoantigens produced by the *plasmodium* apicoplast. Vγ9Vδ2 T cells recognize malaria-derived phosphoantigens such as HMBPP with exquisite sensitivity via a unique mode of presentation [1–5]. Phosphoantigens bind to the conserved transmembrane presenting molecules Butyrophylin 3A1 (BTN3A1) [6] and Butyrophilin 2A1 [7,8], inducing a conformational shift that is sensed via cognate interaction with the Vγ9Vδ2 TCR.

Because BTN3a1 is ubiquitously expressed, this interaction is MHC-unrestricted and does not require professional antigen presenting cells. Thus, Vδ2 T cells act as innate-like effectors that can be rapidly activated during primary infection before an adaptive response has developed. Indeed, massive expansions of Vδ2 T cells have been reported during acute malaria in previously naïve hosts [9–12]. Vδ2 T cells inhibit replication of blood-stage parasites *in vitro* by the release of cytotoxic granules containing granulysin [4,13]. The frequency and malaria-responsiveness of Vδ2 T cells has been shown to correlate with protection from parasitemia in naturally exposed Ugandan children and in malaria-naive volunteers immunized with attenuated *Plasmodium falciparum* sporozoites [14–16].

In contrast to acute malaria, chronic exposure to malaria is associated with a decline in both the frequency of Vδ2 T cells and their ability to produce pro-inflammatory cytokines in response to *P*.*falciparum* antigen. This declining frequency of malaria-reactive Vδ2 T cells has been associated with a lower likelihood of symptoms upon subsequent infection [17]. Chronic malaria exposure results in numerous transcriptional changes in Vδ2 T cells [17]. Among these is expression of the Fc receptor CD16/FcγRIIIa, which is markedly upregulated in the setting of frequent malaria exposure, but is expressed at low levels in children with little or no prior malaria [18]. We have previously shown that CD16/FcγRIIIa expression identifies a subset of Vδ2 T cells that are largely unresponsive to stimulation with *P*.*falciparum* antigen *in vitro* [18].

A notable feature of γδ T cells is their functional plasticity. Prior studies indicate that CD16 discriminates functionally distinct subsets of Vδ2 cells, and that direct ligation of CD16 may provide an alternate pathway of Vδ2 activation [19]. Activation of γδ T cells through CD16 by opsonized antigen has been shown to mediate antibody-dependent cell-mediated cytotoxicity [20], phagocytosis [21], cytokine release [22], and licensing for professional antigen presentation [23,24]. The functional significance of the CD16 signaling pathway in Vδ2 T cells has not been investigated in the setting of malaria, however. We hypothesized that CD16/FcγRIIIa engagement might enable Vδ2 T cells to recognize malaria antigens in chronically malaria-experienced individuals, in cooperation with the humoral immune response.

Here we demonstrate that in heavily malaria-exposed individuals, CD16+ Vδ2 T cells adopt a cytotoxic phenotype and acquire the ability to respond independent of the TCR through engagement of CD16. We found that CD16+ Vδ2 T cells downregulate the TCR and become largely refractory to stimulation with malaria antigen alone, but are able to respond robustly to opsonized malaria antigen, resulting in the release of cytotoxic mediators and inflammatory cytokines. By harnessing the specificity of anti-malarial IgG, this second pathway of activation may enable Vδ2 T cells to mediate anti-parasite immunity in chronically malaria-exposed individuals.

## Methods

### Study site and procedures

Samples were obtained from children and adult caregivers enrolled in a large malaria surveillance survey of two Ugandan districts: the suburban town of Walakuba, Jinja district, with an annual entomological inoculation rate (aEIR) of 2.8, and the rural region of Nagongera, Tororo district, with an aEIR of 310 [25]. Details from this study have been described elsewhere [26]. PBMC were obtained at quarterly routine blood draws. Only timepoints at which the subjects presented without fever or positive blood smear were selected for phenotypic and functional assays. For TCR mean fluorescence intensity staining, additional samples were used from children 36 months of age enrolled in a malaria chemoprevention trial at the Tororo district study site [27].

### Ethical approval

Written informed consent was obtained from all study participants or the guardian of participants under 18 years of age. Study protocols were approved by the Uganda National Council of Science and Technology, the Makerere University School of Medicine Research and Ethics Committee, and the University of California, San Francisco Committee on Human Research.

### Sample processing

6 to 10 milliliters of blood were obtained in acid citrate dextrose tubes. Peripheral blood mononuclear cells (PBMC) were isolated by density gradient centrifugation (Ficoll-Histopaque; GE Life Sciences) and cryopreserved in liquid nitrogen before analysis.

### Malaria antigens

*P*. *falciparum* blood-stage 3D7 parasites were grown in O+ blood by standard methods [28] and harvested at 5–10% parasitemia. Red blood cells infected with mature asexual stages (iRBC) were purified magnetically and cryopreserved in glycerolyte before use. O+ uninfected RBCs (uRBC) were used as controls. Parasites were regularly tested for mycoplasma.

### IgG purification

Total IgG from Ugandan adults and malaria-naïve North American adults was purified from pooled plasma samples using a Pierce protein G agarose column (Thermo Fisher) following standard product protocols (provided with catalog # 20398). Four Ugandan samples were pooled, using 1ml each of plasma. One North American naïve control was used.

### CD16 stimulation

Standard ELISA plates were coated overnight at 4°C with 6μg/well of anti-CD16 antibody (clone 3G8, Biolegend) or Isotype (clone MOPC-21, Biolegend) in 0.1M Carbonate buffer (pH 9.6). Plates were washed twice with PBS and blocked with 200μl/well of RPMI complete media for at least 10 min. 250,000 PBMC were added to each well and the plate was incubated at 37°C for 5 hours prior to surface and intracellular cytokine staining.

### γδ T cell Isolations

γδ T cells were negatively selected from whole PBMC using the Miltenyi human TCRγ/δ+ T cell Isolation Kit (Miltenyi Biotec, catalog# 130092892), following the manufacturer’s protocol with a midiMACS^™^ separator and LS columns.

### iRBC opsonization, BTN3A1 blocking, and stimulation

1x10^6^ iRBC were incubated with 0.5 μg/ml purified IgG from naïve North American controls or hyperimmune Ugandan adults (or media alone) for 1 hour at 37°C in serum free media. For BTN3A1 blocking, PBMC were incubated with 0.5ug/ul of BTN3A1 blocking antibody (clone 103.2, Creative Biolabs) for 30 minutes at room temperature. PBMC and parasites were washed three times in serum free media and combined at a 1:1 effector:target ratio. PBMC and parasites were incubated for 5hr at 37°C prior to surface and intracellular cytokine staining.

### Surface and intracellular cytokine staining

Thawed PBMC were stained for surface markers or rested overnight for *in vitro* stimulation and intracellular cytokine staining. Rested cells were kept in 10% fetal bovine serum (Gibco) and counted before stimulation with either anti-CD16 crosslinking antibody, uRBC, iRBC, or opsonized iRBC. Monensin (Invitrogen/Caltag, 10 μg/ml), Brefeldin-A (Invitrogen/Caltag, 10 μg/ml), and antibody to CD107a were added at the time of stimulation, and stimulation was done at 37°C for 5 hours. Surface and/or intracellular staining was done with standard protocols [29,30] using the antibodies included in S1 Table. KIR expression was investigated only on individuals with known KIR genotypes.

### Flow cytometry data analysis

Flow cytometry profiles were gated on single cells, Aqua Live/Dead negative, CD19-, CD14- lymphocytes that were positive for both CD3 and Vδ2, thus excluding any contribution from NK cells (S1 and S2 Figs). At least 100,000 events were collected. Prior phenotypic work revealed that over 80% of peripheral blood Vδ2 T cells also express Vγ9 [18], thus IFNγ and CD107a expression was quantified using only Vδ2. Instrument settings were normalized across runs with SPHERO Rainbow Calibration Particles (Becton Dickinson) to ensure the validity of MFI comparisons. Samples were analyzed on an LSR2 flow cytometer (Becton Dickinson) with FACSDiva software. Data were analyzed using FlowJo (Tree Star).

### Immunofluorescence microscopy

For immunofluorescence analysis, iRBCs were mixed with uRBC at a 1:1 ratio, incubated with naïve IgG or hyperimmune IgG for 1 hr at 37°C, washed three times in serum free media, then washed an additional three times in PBS with 5% FBS. iRBC were stained with anti-human IgG Alexa 488 (ThermoFisher) at a 1:400 dilution for 30 min at room temperature, then fixed in 4% PFA for 20 min. DAPI mounting media (ProLong Gold, ThermoFisher) was used to visualize the *P*.*falciparum* DNA. Immunofluorescence images were obtained on a Keyence BZ-X710.

### Statistical methods

All statistical analyses were performed using Prism 6.0 (GraphPad), and/or STATA version 13 (StataCorp). Comparisons of phenotypic markers between CD16+ and CD16- Vδ2 T cells and of cytokine production and degranulation between stimulation conditions were performed using the Wilcoxon matched-pairs signed-rank test. Grouped scatter plots show medians and interquartile range, and box plots show medians with Tukey whiskers. Continuous variables were compared using Spearman correlation. Scatter plots show best fit linear regression lines with 95% confidence intervals. In all analyses, a 2-tailed P value <0.05 was considered significant.

## Results

### CD16+ Vδ2 T cells exhibit a cytotoxic phenotype

Vδ2 T cells demonstrate considerable cytolytic capacity against tumor cells and microbially-infected cells [31,32]. To determine whether the upregulation of CD16 observed in individuals with chronic malaria infections is associated with enhanced cytotoxic potential, we assessed adults and children residing in both high and low malaria transmission areas for Vδ2 T cell expression of a variety of proteins associated with cytotoxicity in T cells. These included the effector molecules granzyme B, granulysin, and perforin, as well as CX3CR1, a fractalkine receptor that identifies cytotoxic subsets of CD8, CD4 and NK cells [33,34], and the transcription factors Tbet and Eomes.

**Fig 1:**
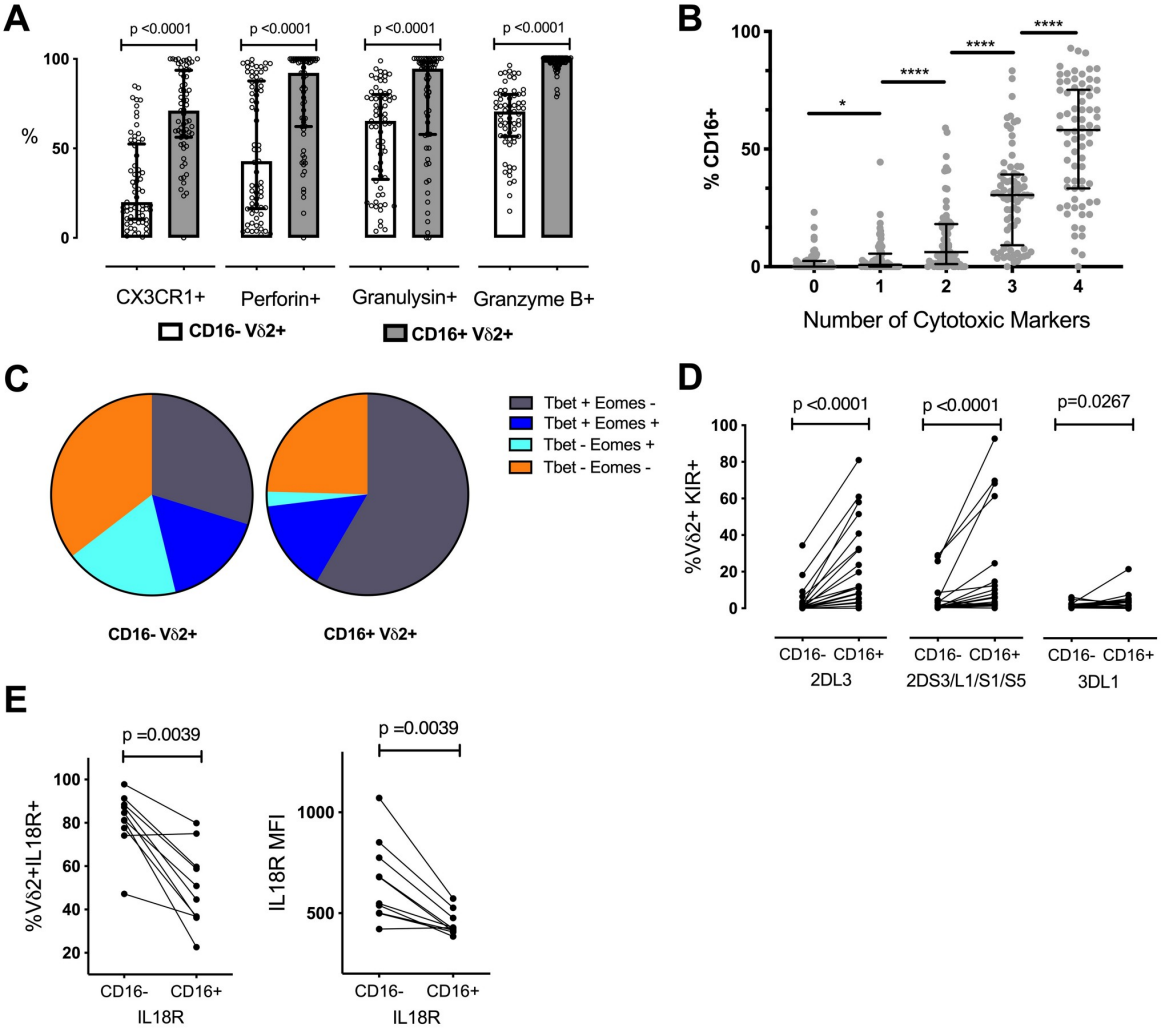
CD16+ Vδ2 T cells are more likely to express cytotoxic markers and KIRs. (A) The percentage of CD16+ and CD16-Vδ2 T cells expressing relevant cytotoxic markers are shown (n = 65; scatter plot with median and IQR). (B) Vδ2 T cells positive for 4 or any combination of 3, 2 or 1 of the cytotoxicity markers listed in A are grouped along the x-axis and their relative percentage of CD16 is shown (n = 65; scatter plot with median and IQR). (C) The percentage of CD16+ and CD16-Vδ2s expressing the listed combinations of the transcription factors Tbet and Eomes are shown. (n = 65). (D) The percentage of CD16+ and CD16-Vδ2 T cells expressing the listed KIR or KIR combinations is shown. Individuals who were genotype negative for the listed KIRs were omitted from this analysis (n = 22 for KIR3DL1 and KIR2DS3/L1/S1/S5; n = 20 for KIR2DL3). (E) The percentage of CD16+ and CD16-Vδ2 T cells expressing IL18Rα and the MFI of IL18Rα on CD16+ and CD16-Vδ2 T cells is shown (n = 10). All Vδ2+ T cells shown were gated on singlets and CD3+ events to exclude NK cells. P values for A, B, D and E determined by Wilcoxon matched pairs signed rank test.

The proportion of CD16+ Vδ2 T cells expressing CX3CR1, granulysin, perforin, and granzyme B was significantly higher than in CD16- Vδ2 T cells (all p<0.0001; Fig 1A). This pattern was observed in all age groups and in both high and low malaria transmission areas (S3 Fig). Additionally, the more cytotoxic markers expressed by an individual Vδ2, the more likely it was to be CD16+ (Fig 1B), indicating that CD16 expression is associated with an increasingly complex cytotoxic phenotype rather than the expression of any one effector molecule. The majority of CD16+ Vδ2 T cells were also Tbet+ and Eomes–(Fig 1C), a transcription factor profile linked with cytotoxicity and lack of long term memory formation in CD8 T cells [35,36], and with terminally differentiated, cytotoxic CD56^dim^CD16^hi^ NK cells [37].

Additionally, we measured Vδ2 expression of killer cell immunoglobulin-like receptors (KIR), a family of activating and inhibitory receptors commonly associated with CD56dimCD16+ NK cells. For each of the three distinct KIR groups tested (KIR2DL3, KIR2DL1/S1/S3/S5, and KIR3DL1), CD16+ Vδ2 T cells were more likely than CD16- Vδ2 T cells to express KIR (Fig 1D). Because KIR-expressing CD56dimCD16+ and adaptive NK cells are generally less responsive to inflammatory cytokines [38], we also measured the expression of receptors for NK cell accessory cytokines such as IL2R/CD25, IL12R, IL15R and IL18R on Vδ2 T cells in a subset of high exposure and low exposure individuals. Little to no expression of IL2R, IL12R or IL15R was observed on Vδ2 T cells, either *ex vivo* or after stimulation with HMBPP or PMA/Ionomycin. IL18R was highly expressed, but both the frequency of expression and surface density of IL18R were markedly lower on CD16+ Vδ2 T cells (Fig 1E). This difference is consistent with parallels between CD16+ Vδ2 T cells and highly differentiated NK cells. Together, these data suggest that in the wake of frequent malaria exposure, Vδ2 T cells acquire cytotoxic functions and adopt an NK-like phenotype along with CD16 expression, and that this pattern is consistent across different age strata and transmission zones.

### CD16+ Vδ2 T cells from malaria-exposed individuals down-regulate TCR and can be directly activated through CD16

**Fig 2:**
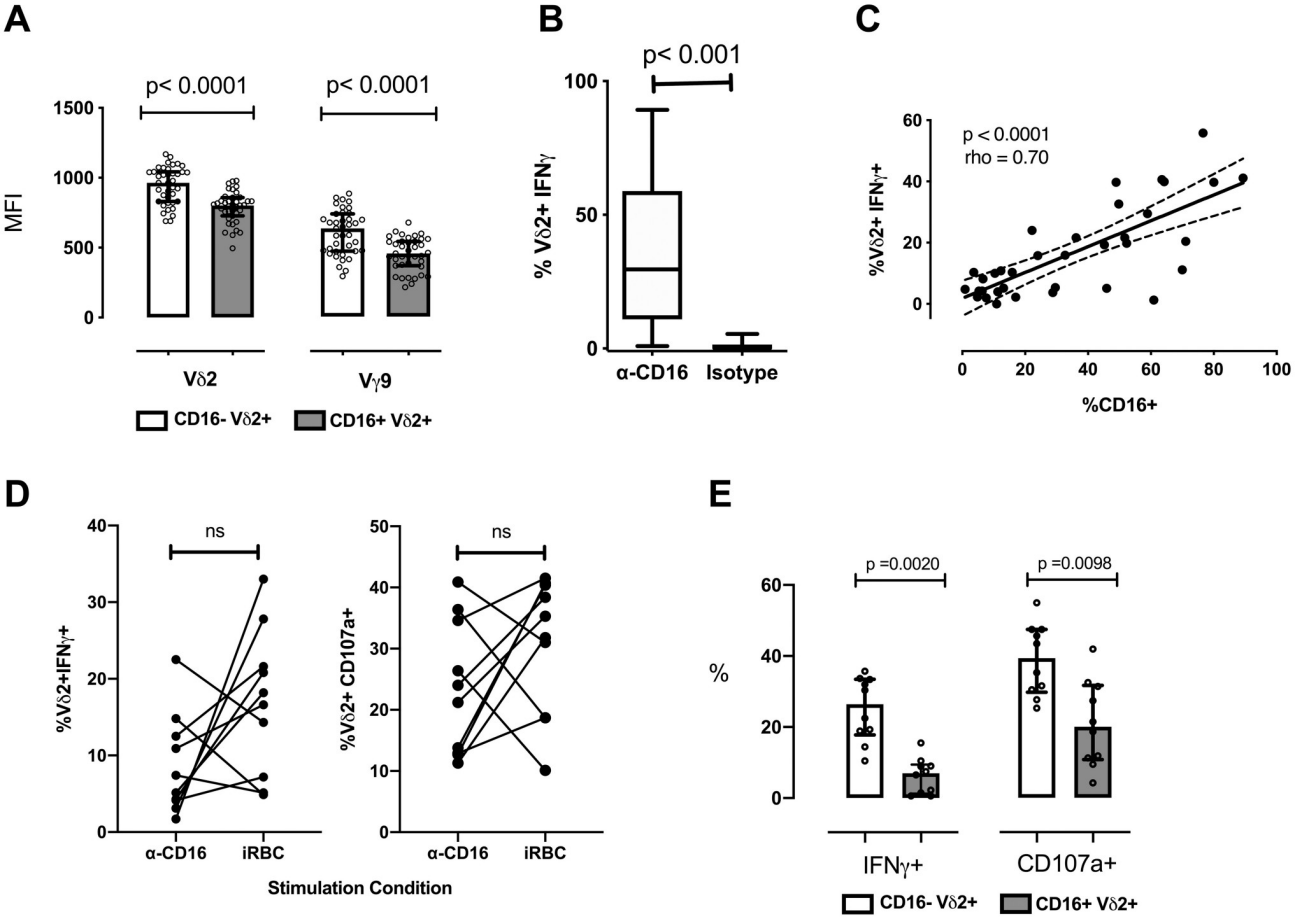
CD16+ Vδ2 T cells from malaria-exposed individuals downregulate TCR and can be independently stimulated through CD16. (A)The MFI of the Vδ2 and Vγ9 chains is shown for CD16+ and CD16- Vδ2+Vγ9+ T cells. Data points and accompanying p-value are paired. (n = 39; scatter plots with median and IQR). (B) The percentage of Vδ2 T cells producing IFNγ in response to anti-CD16 crosslinking antibody or isotype is compared (n = 35; box plots with median and Tukey whiskers). (C) The percentage of Vδ2 T cells producting IFNγ in response to plate-bound anti-CD16 crosslinking antibody is graphed against the percentage of Vδ2 T cells expressing CD16 prior to stimulation (n = 35). (D) The percentage of Vδ2 T cells positive for IFNγ or CD107a after stimulation with anti-CD16 crosslinking antibody or iRBC is compared (n = 10) (E) Vδ2 T cells positive for IFNγ or CD107a after iRBC stimulation from the experiment in E are grouped by CD16 expression. All Vδ2+ T cells shown were gated on singlets and CD3+ events to exclude NK cells. P values determined by Wilcoxon matched pairs signed rank test in A, B, D and D, and with Spearman correlation in C.

We have previously shown that the frequency of Vδ2 T cells expressing CD16 is elevated in children heavily exposed to malaria, and that CD16+ Vδ2 T cells are generally unresponsive to *P*.*falciparum* antigen stimulation *in vitro* [18]. To investigate the mechanism for this reduced reactivity to *P*.*falciparum*-derived phosphoantigens, we compared expression levels of the γδ TCR on CD16+ and CD16- Vδ2 T cells. We found that both the γ and δ chains of the TCR were strikingly downregulated on Vδ2 T cells that had acquired CD16 expression (both p<0.0001; Fig 2A).

A few studies have shown that Vδ2 T cells can be activated by CD16 crosslinking and/or opsonized viral antigen to mediate effector functions [19,22], similar to the antibody-dependent functions of NK cells. We therefore hypothesized that CD16 functions as an alternative activating receptor on Vδ2 T cells from chronically malaria-exposed individuals who respond poorly to parasite antigen alone [18]. To test this, we incubated Ugandan PBMC with plate-bound CD16-crosslinking antibody and measured production of IFNγ. Vδ2 T cells from all individuals were able to produce IFNγ in response to CD16 crosslinking (Fig 2B). The percentage of IFNγ-producing Vδ2 T cells was variable among subjects and strongly correlated with the percentage of CD16+ Vδ2 T cells present in each sample (Fig 2C). Overall, the proportion of Vδ2 T cells that degranulated (measured as the mobilization of LAMP1/CD107a) or produced IFNγ in response to CD16 engagement was comparable to, and in some cases exceeded, the proportion responding to *P*.*falciparum*-infected red blood cells (iRBC) (Fig 2D). This is likely due to the fact that, as we have previously shown [18], CD16+ Vδ2 T cells are largely unresponsive to iRBC stimulation (Fig 2E). Together, these results demonstrate that Vδ2 T cells that are refractory to stimulation by malaria antigen alone can nonetheless produce IFNγ and degranulate when activated through CD16, suggesting a potential physiologic role for antibody engagement in the activation of Vδ2 cells.

### Stimulation through CD16 augments Vδ2 activation by *P*. *falciparum*

**Fig 3:**
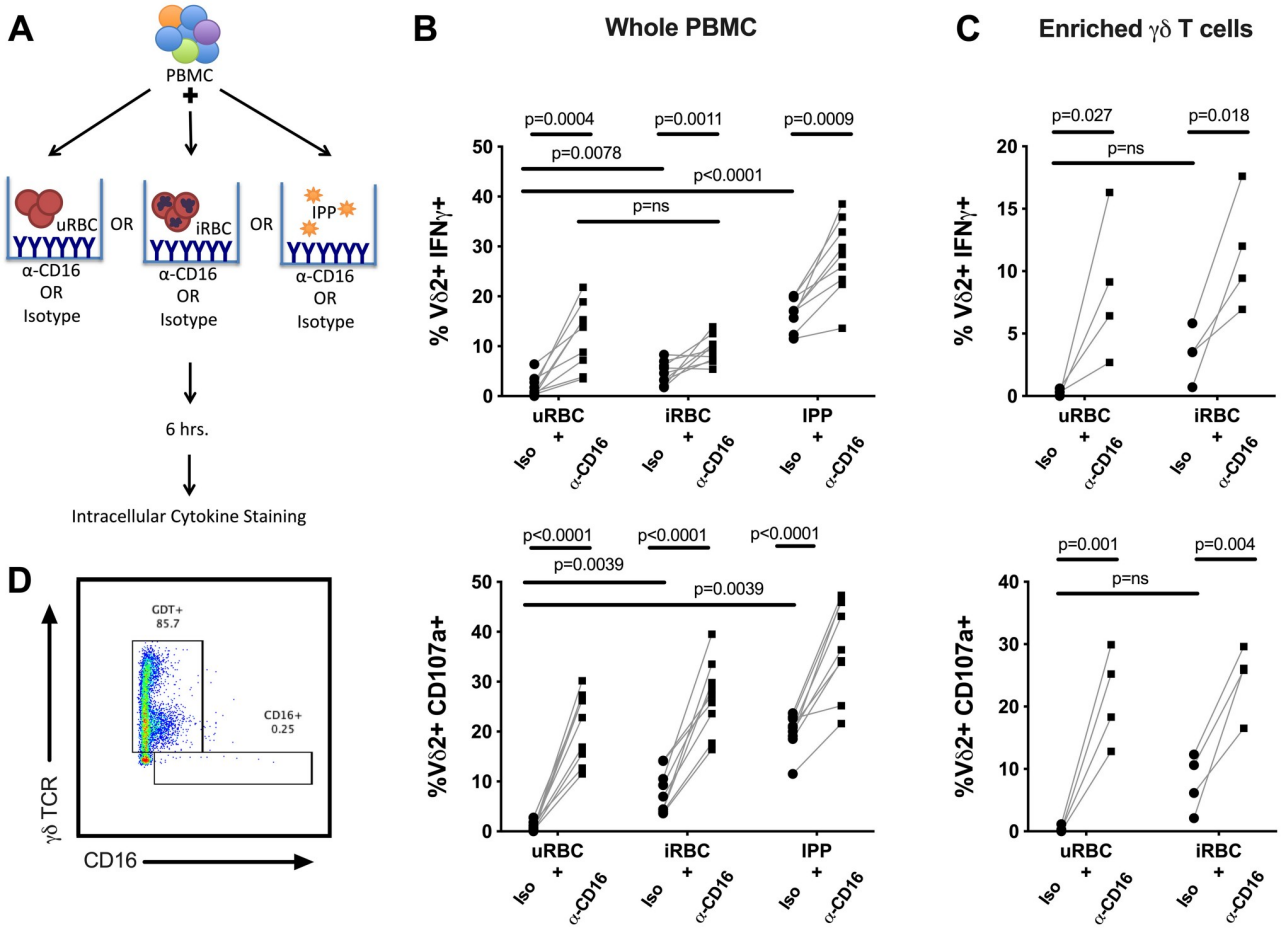
Stimulation through CD16 augments Vδ2 cell activation by *P*.*falciparum*. (A) Schematic detailing stimulation conditions for data presented in panel B. (B) Percent of Vδ2 T cells producing IFNγ or positive for CD107a after stimulation of whole PBMC with uRBC, iRBC or IPP, with or without plate bound anti-CD16 crosslinking antibody (n = 9). (C). Percent of Vδ2 T cells producing IFNγ or positive for CD107a after stimulation of negatively selected γδ T cells with uRBC or iRBC, with or without plate bound anti-CD16 crosslinking antibody (n = 4). (D) Live cells positive for γδ TCR after negative selection. All Vδ2+ T cells shown in B-C were gated on singlets and CD3+ events to exclude NK cells. Samples for these experiments were selected from individuals with greater than 30% of their Vδ2 T cells expressing CD16. P values were determined by Wilcoxon matched pairs signed rank test.

To investigate the physiologic relevance of CD16-mediated Vδ2 effector functions in malaria infection, we combined iRBC stimulation of PBMC with the CD16 crosslinking assay used above (Fig 3A) to determine whether CD16+ Vδ2 T cells can be activated alternately or synergistically through CD16, rather than via TCR alone. We observed an increase in both IFNγ production and degranulation in the presence of CD16 crosslinking antibody, compared with iRBC plus isotype control (Fig 3B). A similar increase in activation was observed when the phosphoantigen isopentenyl pyrophosphate (IPP) was used to stimulate the Vδ2 TCR, further suggesting that additional Vδ2 T cells are activated through CD16 ligation. To ensure that this increased activation was not simply a bystander effect of cytokines released by other innate CD16+ cells (NK cells, monocytes), we repeated these experiments with enriched γδ T cells isolated via negative selection, achieving a purity of approximately 85% with 0.25% CD16+ γδ TCR- cells remaining (Fig 3D). Vδ2 T cells from these highly enriched populations displayed a similar increase in IFNγ and degranulation in the presence of CD16 crosslinking antibody over iRBC alone, indicating that direct contact of Vδ2 T cells with CD16 was likely responsible for this effect (Fig 3C).

**Fig 4:**
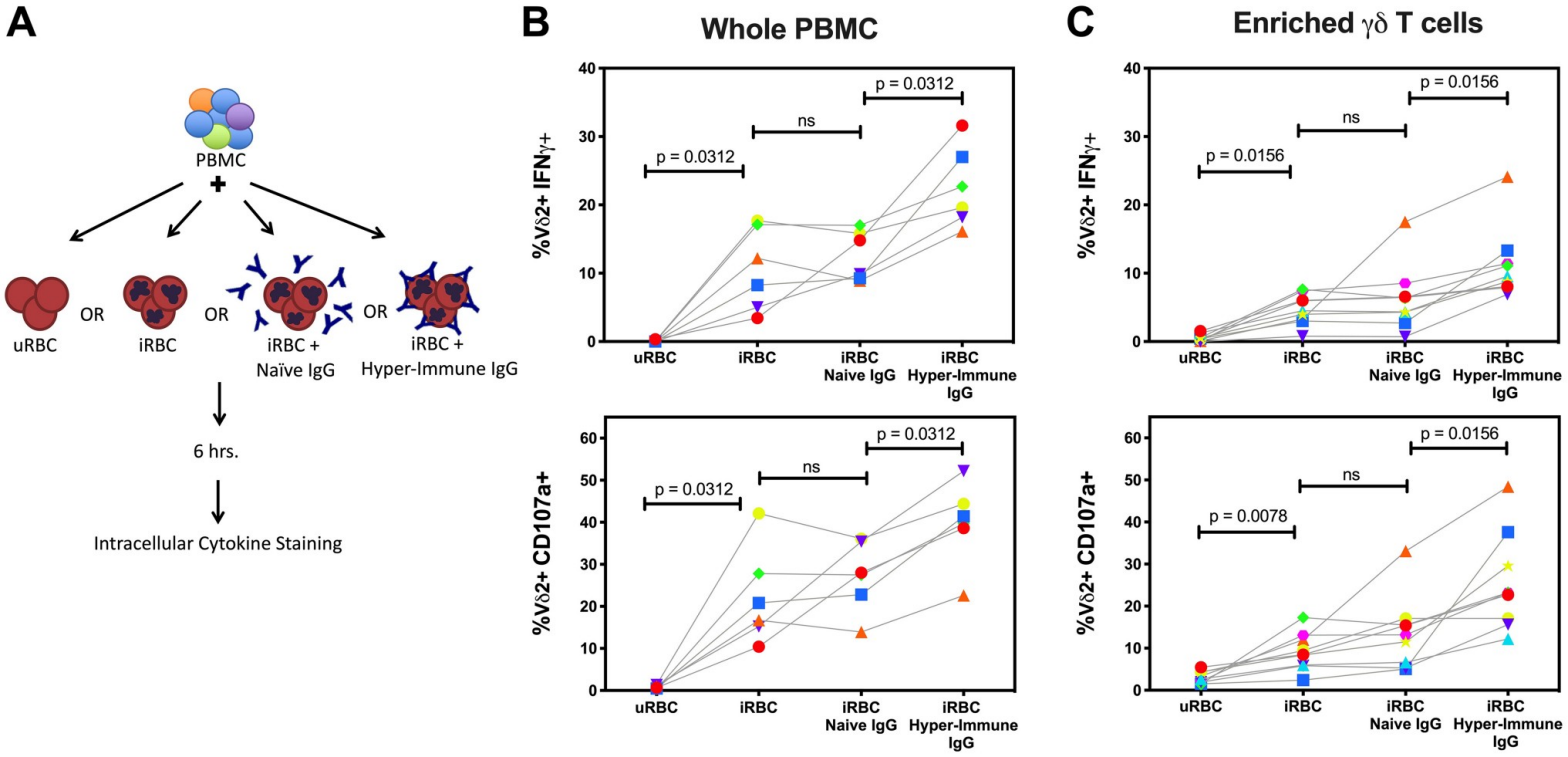
Stimulation by opsonized antigen augments Vδ2 cell activation by *P*.*falciparum*. (A) Schematic detailing the stimulation conditions for data presented in panel B. (B) Percent of Vδ2 T cells positive for IFNγ and CD107a after stimulation of whole PBMC with uRBC, iRBC, iRBC + naïve IgG, or iRBC + hyper-immune IgG. (n = 6). (C) Percent of Vδ2 T cells positive for IFNγ and CD107a after stimulation of negatively selected γδ T cells with uRBC, iRBC, iRBC + naïve IgG, or iRBC + hyper-immune IgG. (n = 9). All Vδ2+ T cells shown were gated on singlets and CD3+ events to exclude NK cells. Samples for this experiment were selected from individuals with greater than 30% of their Vδ2 T cells expressing CD16. P values were determined by Wilcoxon matched pairs signed rank test.

Hypothesizing that CD16 might enable Vδ2 T cells to recognize opsonized parasite antigen, we purified IgG from malaria-naïve individuals and from adults living in a highly malaria endemic region of Uganda in order to opsonize parasites in culture. Fluorescent microscopy confirmed co-localization of this “hyperimmune” IgG with lab grown parasites (S4 Fig). We then compared cytokine production and degranulation between Vδ2 T cells stimulated with iRBC in the presence of hyperimmune IgG or IgG purified from malaria-naive adults (Fig 4A). The addition of hyperimmune (but not naïve) IgG led to a significant increase (4–20% more activated Vδ2+ events) in the percentage of Vδ2 T cells that degranulated and produced IFNγ (Fig 4B). This was true for both whole PBMC and for highly enriched γδ T cells (Fig 4C), indicating that the increase in Vδ2 T cell stimulation observed with hyper-immune IgG was not merely a bystander effect of cytokines released by monocytes or NK cells engaged by opsonized antigen. Together these experiments indicate that opsonized antigen augments the activation of Vδ2 T cells through CD16 engagement, either independent of or in combination with conventional TCR simulation.

As further evidence that the CD16 activation pathway was triggered by opsonized antigen, surface expression of CD16 was lost following stimulation with iRBC in the presence of hyperimmune IgG, but not naïve IgG (S5A Fig). This is consistent with published data demonstrating that after engagement, CD16 is cleaved from the surface of NK cells and T cells by the metalloprotease ADAM17 [39]. Because of this activation-induced CD16 shedding, we were unable to stain directly for CD16 on Vδ2 T cells after stimulation to confirm that these were the responding cells. Instead, as a surrogate of CD16- Vδ2 T cells, we identified cells positive for CD28, as Ryan et al. [40] have shown that Vδ2 cell expression of CD16 and CD28 are mutually exclusive. As anticipated, we observed almost no CD28+CD16+ events pre-stimulation (S5B Fig), nor was CD28 expression altered post-stimulation. Following stimulation with iRBC and hyperimmune IgG, IFNγ production was enhanced in the CD28- Vδ2 subset much more so than in CD28+ (putative CD16-) Vδ2 T cells. This further supports the conclusion that the CD28-CD16+ subset of Vδ2 T cells can be specifically activated by opsonized malaria antigen.

### The CD16-mediated Vδ2 cell response to opsonized antigen is independent of the TCR

**Fig 5:**
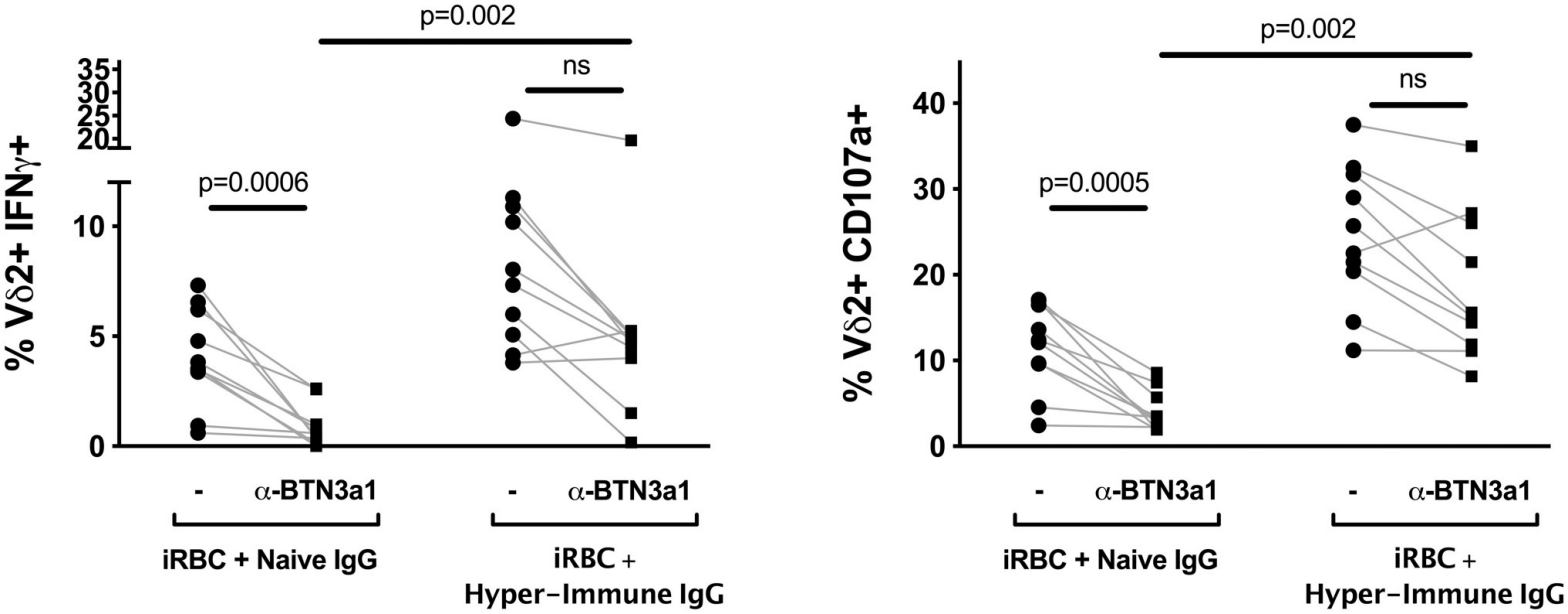
Blocking BTN3a1 does not inhibit stimulation of Vδ2 T cells by opsonized antigen. Percent of Vδ2 T cells positive for IFNγ and CD107a after stimulation with iRBC + naïve IgG, or with iRBC + hyper-immune IgG, are compared in the presence or absence of a Butyrophilin 3a1 blocking antibody. (n = 10; p values determined by Wilcoxon matched pairs signed rank test). All Vδ2+ T cells shown were gated on singlets and CD3+ events to exclude NK cells. Samples for this experiment were selected from adults with greater than 30% of their Vδ2s expressing CD16.

Finally, to determine whether stimulation of Vδ2 T cells via CD16 requires concomitant engagement of the TCR, we incubated PBMC with an antibody that blocks the butyrophilin presenting molecule BTN3a1, to eliminate the presentation of phosphoantigens to the Vδ2Vγ9 TCR. PBMC were then stimulated with iRBC in the presence of either naïve or hyperimmune IgG, as before. When only naïve IgG was present, BTN3a1 blockade diminished IFNγ production and degranulation to near background levels (Fig 5). However, when hyperimmune IgG was present, significant IFNγ production and degranulation was observed even in the presence of BTN3a1 blockade (p = 0.002 for naïve vs. hyperimmune IgG), indicating that Vδ2 effector functions can be activated by CD16 engagement alone, independent of the TCR.

## Discussion

**Fig 6:**
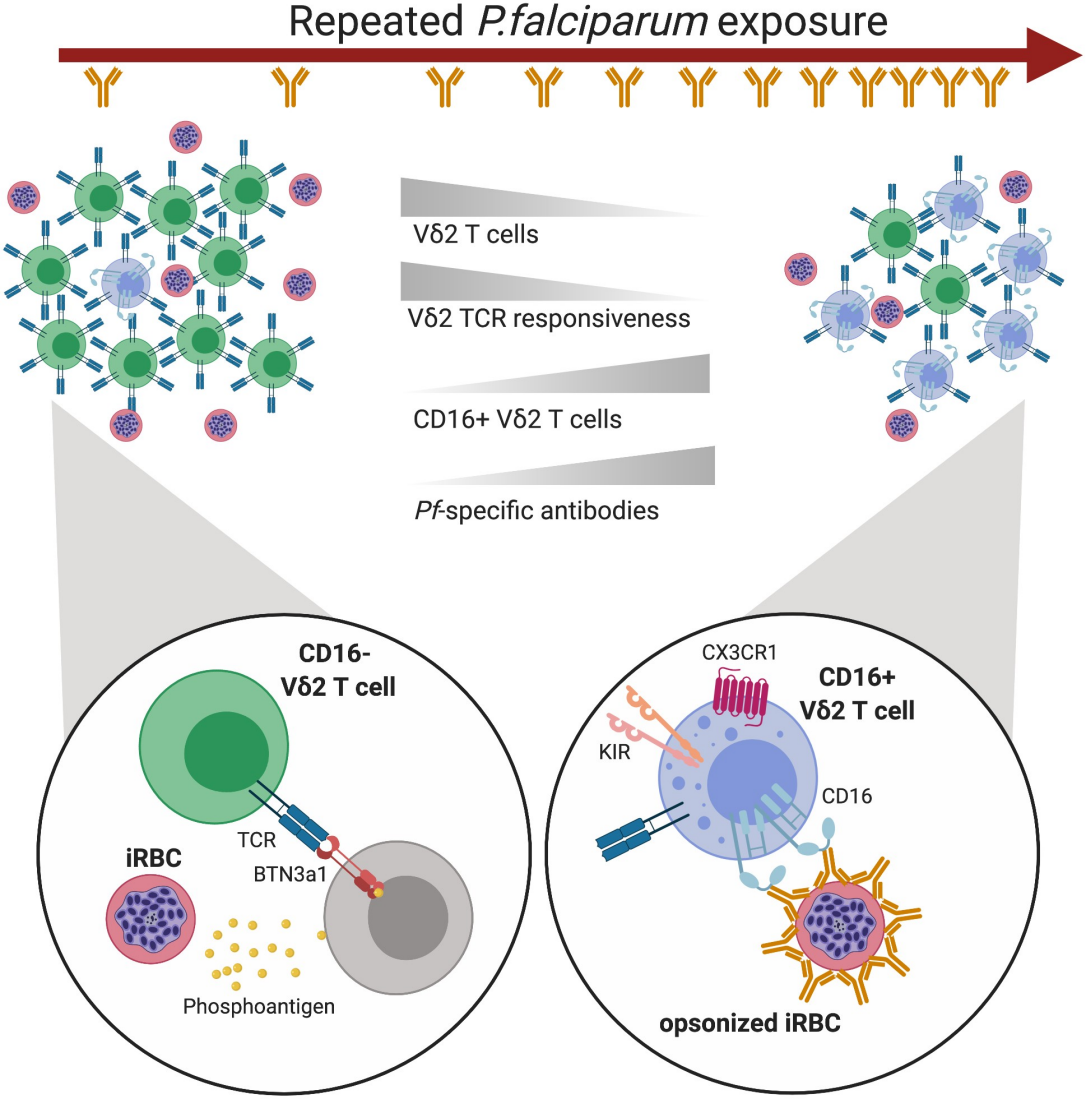
Model of Vδ2 T cells over the course of repeated *Pf* infection. As an individual experiences more episodes of *Pf*, the frequency of Vδ2 T cells in their peripheral blood declines. Remaining Vδ2 T cells become less responsive through their TCRs while the frequency of CD16+ expression increases. These changes occur while an individual’s anti-*Pf* antibody response is simultaneously growing and diversifying, presumably providing more opsonized parasite targets for CD16+ Vδ2 T cells. TCR-responsive Vδ2s are activated by engagement with BTN3A1 on another cell, probably a lymphocyte, when BTN3A1’s conformation is altered by the intracellular binding of parasite-derived phosphoantigens. CD16-responsive Vδ2 T cells are activated by opsonized parasite antigen and may have their activation threshold modulated by KIR expression and engagement. Created with Biorender.

Our results show that *P*. *falciparum* can activate Vδ2 cell effector functions via two distinct pathways. The first, previously characterized pathway is via TCR-mediated recognition of phosphoantigens produced by the *plasmodium* apicoplast. This response is attenuated with repeated malaria antigen exposure, likely due to downregulation of the γδ TCR. Here, we show that opsonized *P*. *falciparum* parasites can stimulate a sizable subset of Vδ2 T cells directly through CD16, independent of TCR. This is the first work to demonstrate a functional role for CD16 on γδ T cells in malaria infection. Additionally, these results broaden the potential role for opsonizing IgG in antimalarial immunity (Fig 6).

Vδ2 T cells are increasingly recognized as an important component of the cellular immune response to malaria. In a clinical trial of sporozoite vaccination, the frequency of unstimulated Vδ2 T cells both before and after immunization correlated with protection from challenge by controlled human malaria infection, while the frequency of *P*.*falciparum-*specific CD4 T cells did not [14]. In endemic settings, higher frequencies of Vδ2 cells, as well as higher percentages of Vδ2 T cells that produce IFNγ and TNF upon malaria antigen stimulation, have been associated with protection against parasitemia [16].

In healthy, malaria-naïve individuals, Vδ2 T cells comprise up to 5% of the T cell population in peripheral blood, the majority of which display an effector memory phenotype and respond without prior activation [41]. Thus, Vδ2 T cells are capable of an immediate innate-like effector response with the release of inflammatory cytokines that may be crucial to controlling parasite replication in a malaria-naïve host. However, continued release of inflammatory cytokines could result in tissue damage and/or severe malaria [42], and parasite tolerance may be achieved, in part, by a reduction in the number of Vδ2 T cells capable of responding to phosphoantigen. Indeed, we have shown that with increasing malaria exposure, Vδ2 T cells decline in the peripheral blood, upregulate immunoregulatory markers, and become unresponsive to iRBC [17,18].

The experiments presented here demonstrate that Vδ2 T cells previously thought to be dysfunctional or exhausted due to repeated TCR stimulation can instead be activated to mediate cytolytic effector functions through CD16, harnessing the specificity of anti-malarial IgG antibodies. This expands the role for opsonizing antibody in antimalarial immunity. These results are in line with the growing recognition that cooperation between the cellular and humoral arms of the immune system is necessary for an optimal immune response, and suggest that immunity in repeatedly exposed individuals may be fine-tuned by Fc features such as IgG isotype, subclass, and glycosylation [43]. Notably, CD16+ Vδ2 T cells from malaria-exposed individuals phenotypically resemble mature NK cells, including expression of the cytolytic effector molecules perforin, granzyme B and granulysin, as well as CX3CR1 and KIRs, and down-regulation of IL18R. We show that CD16+ Vδ2 T cells resemble NK cells not only phenotypically, but also functionally, using flow cytometry assays gated specifically on Vδ2+ T cells (excluding NK cells) to demonstrate degranulation and cytokine production in the presence of hyperimmune serum. Hence, the role of CD16+Vδ2 T cells antigens may be functionally redundant to that of other FcR+ lymphocytes such as NK cells in recognizing IgG-opsonized malaria antigens, or perhaps cytotoxic CD16+Vδ2 T cells are tailored toward particular anatomic compartments, such as the microvasculature.

Our results further underscore that γδ T cells are capable of remarkable functional plasticity. In addition to ADCC and cytokine release, Vδ2 T cells have been reported to participate in phagocytosis and antigen presentation [21,23,24,44]. Following stimulation in culture, Vδ2 T cells transition from a predominately inflammatory, cytokine-producing state to one capable of phagocytosing opsonized *E*. *coli*, while also upregulating HLA-DR and CD86. Because *E*. *coli* are similar in size to merozoites, the extracellular blood stage of *P*.*falciparum* parasites, one could easily postulate that Vδ2 T cells expressing CD16 are also able to phagocytose opsonized merozoites. There is also evidence that Vδ2 T cells can process and present antigen to CD8 and CD4 T cells [23,24], including during malaria infection [45]. Thus, Vδ2 T cells may play a unique multi-faceted role in the anti-malarial T cell response.

Finally, our data indicate that peripheral blood Vδ2 T cells are not homogeneous, but instead comprise a complex array of phenotypically and functionally distinct subsets. Vδ2 T cells with Th1-like, Th2-like, Th17-like, and Treg effector characteristics have all been described [46]. Consistent with our observations, a recent transcriptional analysis of Vδ2 T cells from malaria-naïve individuals found polarization toward two dominant phenotypes: one, CD28-CD16+, with greater degranulation potential, and another, CD28+CD16-, with greater proliferative potential and increased chemokine receptor expression [40]. In heavily malaria-exposed populations, the shift toward CD16+ Vδ2 T cells may indicate the increasing importance of Fc-mediated degranulation and possibly phagocytosis in the chronic phase of malaria infection (Fig 5). Substantial heterogeneity can be observed within the Vδ2 population of a chronically exposed individual, with some (especially CD16-negative) Vδ2 cells remaining phosphoantigen-reactive, but an increasing proportion downregulating the γδTCR and acquiring responsiveness to CD16 stimulation.

This study had several limitations. Due to the low prevalence of Vδ2 T cells in peripheral blood, particularly in chronically malaria-exposed individuals, we were not able to sort a sufficient number of unstimulated CD16- and CD16+ Vδ2 T cells for quantitative *in vitro* growth inhibition assays. Additionally, because CD16 is cleaved after crosslinking, we were unable to discriminate the CD16+ Vδ2 cell population by flow cytometry after stimulation. We did observe a dramatic decline in surface CD16 expression following stimulation with hyperimmune IgG (but not naïve IgG), indicating that the response was, indeed, mediated by CD16 crosslinking. However, we cannot exclude the possibility that other Fc receptors may also play a role in the Vδ2 response to opsonized antigen.

Lastly, the hyperimmune IgG used to opsonize parasites is poly-specific and presumably contains antibodies specific for non-malaria antigens, including potential human cell-associated proteins (e.g. blood group antigens or erythrocyte-reactive antibodies). Additionally, there is evidence that malaria infection can lead to the production of erythrocyte-reactive auto-antibodies that contribute to malarial anemia [47,48]. Thus, a minority of Vδ2 T cells in our experiments may have been activated by IgG bound to non-malarial antigens.

In summary, we show that Vδ2 cells, which are already known to play an important role as innate-like effectors via their TCR-mediated functions, can be alternatively activated via the Fc receptor CD16 which is upregulated with chronic malaria exposure. These findings suggest a new role for CD16+ Vδ2 cells, in partnership with opsonizing antimalarial antibodies, in controlling *P*.*falciparum* infections of heavily exposed individuals.

## Supporting information

S1 Table Antibody details. (TIF)

S1 Fig Gating schematic for phenotypic assays. Vδ2 T cells evaluated for phenotypic markers of cytotoxicity were gated first by comparing FSC area to FSC height to exclude doublets, then by using FSC and SSC to select lymphocytes, then by excluding DUMP (CD14, CD19, Aqua live/dead) positive cells, and finally by selecting for CD3 positive and Vδ2 positive events. Fluorescence minus one (FMO) controls were used to define the gates for CD16+, GranzymeB+, Perforin+, Granulysin+, CX3CR1+, Eomes+, and Tbet+ events, as shown.
(TIF)

S2 Fig Gating schematic for functional assays. Vδ2 T cells evaluated for cytokine expression and degranulation after *in vitro* stimulation were gated first by comparing FSC area to FSC height to exclude doublets, then by using FSC and SSC to select lymphocytes, then by excluding DUMP (CD14, CD19, Aqua live/dead) positive cells, and finally by selecting for CD3 positive and Vδ2 positive events. uRBC or isotype controls were used to define IFNγ and CD107a positive events, as shown.
(TIF)

S3 Fig CD16+ Vδ2 T cells are more likely to express cytotoxic markers regardless of age and exposure history. (A) The percentage of CD16+ and CD16-Vδ2 T cells expressing relevant cytotoxic markers, divided by age (children vs adults) and exposure history (high exposure = Nagogera, low exposure = Walakuba) (Walakuba children, n = 20; Nagongera children, n = 20; Walakuba adults, n = 13; Nagongera adults, n = 12). Scatter plots with median and IQR are shown. P values were determined by Wilcoxon matched pairs signed rank test.
(TIF)

S4 Fig Hyper-immune IgG opsonizes 3D7 *P*.*falciparum*-infected RBCs. (A) Representative fluorescent images from the coincubation of iRBC with purified immune or naïve IgG (B) Quantification of the number of opsonized events per field (n = 20) in A.
(TIF)

S5 Fig CD16 is lost from Vδ2 T cells following stimulation with opsonized antigen, yet CD28-Vδ2 T cells are responsible for the increase in stimulation attributed to opsonized antigen. (A) Representative plot of CD16 vs CD28 expression on Vδ2 T cells after stimulation with iRBC+ naïve IgG or iRBC + hyper-immune IgG (B) Percent of Vδ2 T cells expressing CD16 or CD28 after the stimulation conditions listed. (C) The percent increase in IFNγ and CD107a+ Vδ2 T cells after stimulation with opsonized antigen (iRBC+ hyperimmune IgG) vs. unopsonized antigen (iRBC + naïve IgG), divided by CD28 expression (n = 6; p values determined by Wilcoxon matched pairs signed rank test).
(TIF)

S1 Data Values used to construct all graphs in the manuscript are listed in tables, labeled with their corresponding figure number and panel. (XLSX)
