# Supplementary figures and images for: Opsonized antigen activates Vδ2+ T cells via CD16/FCγRIIIa in individuals with chronic malaria exposure
Source: PLoS Pathog. 2020 Oct 21;16(10):e1008997. doi: 10.1371/journal.ppat.1008997 (PMC7605717; doi:10.1371/journal.ppat.1008997)

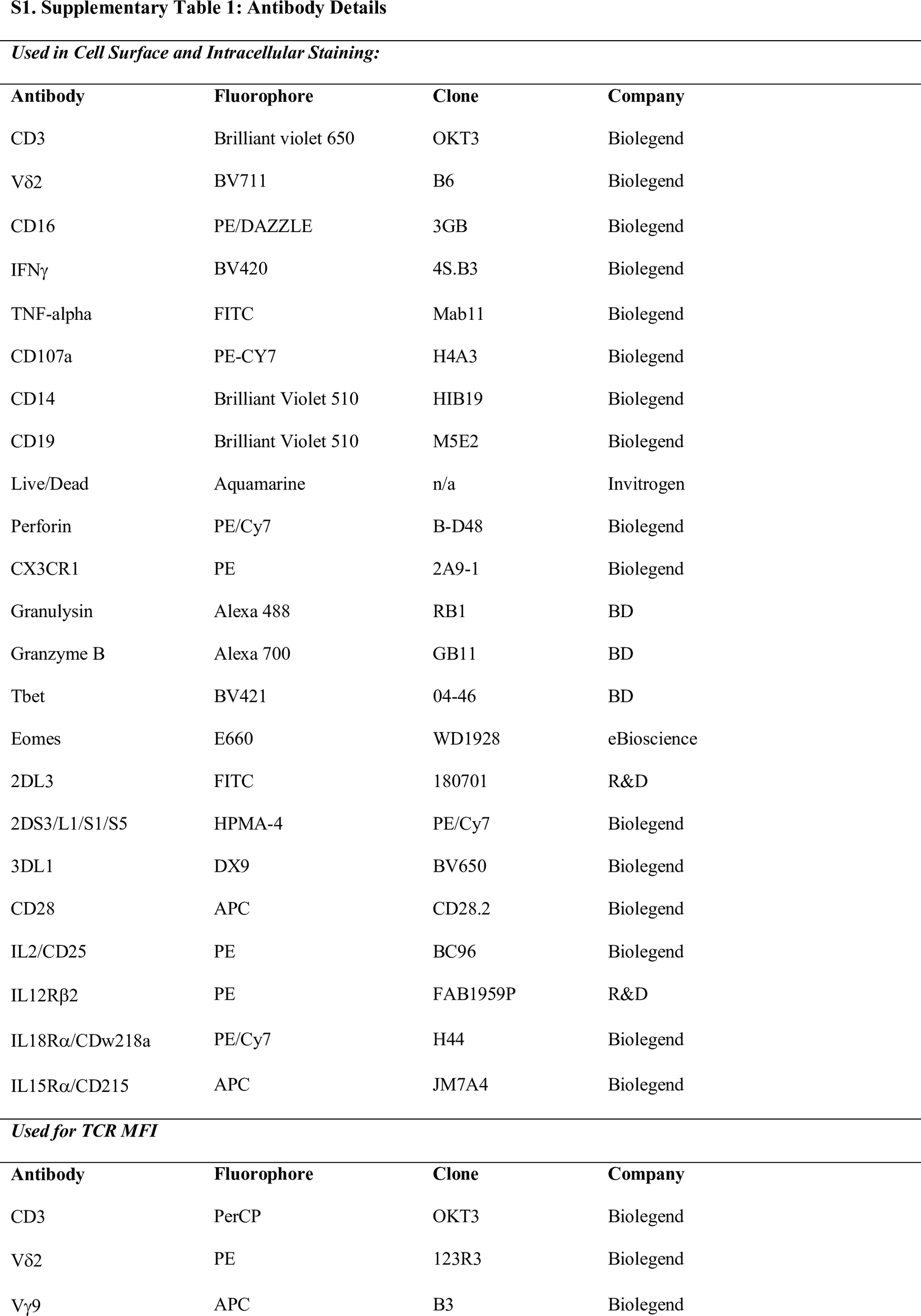

Supplement: S1 Table — (TIF) [file ppat.1008997.s001.tif]

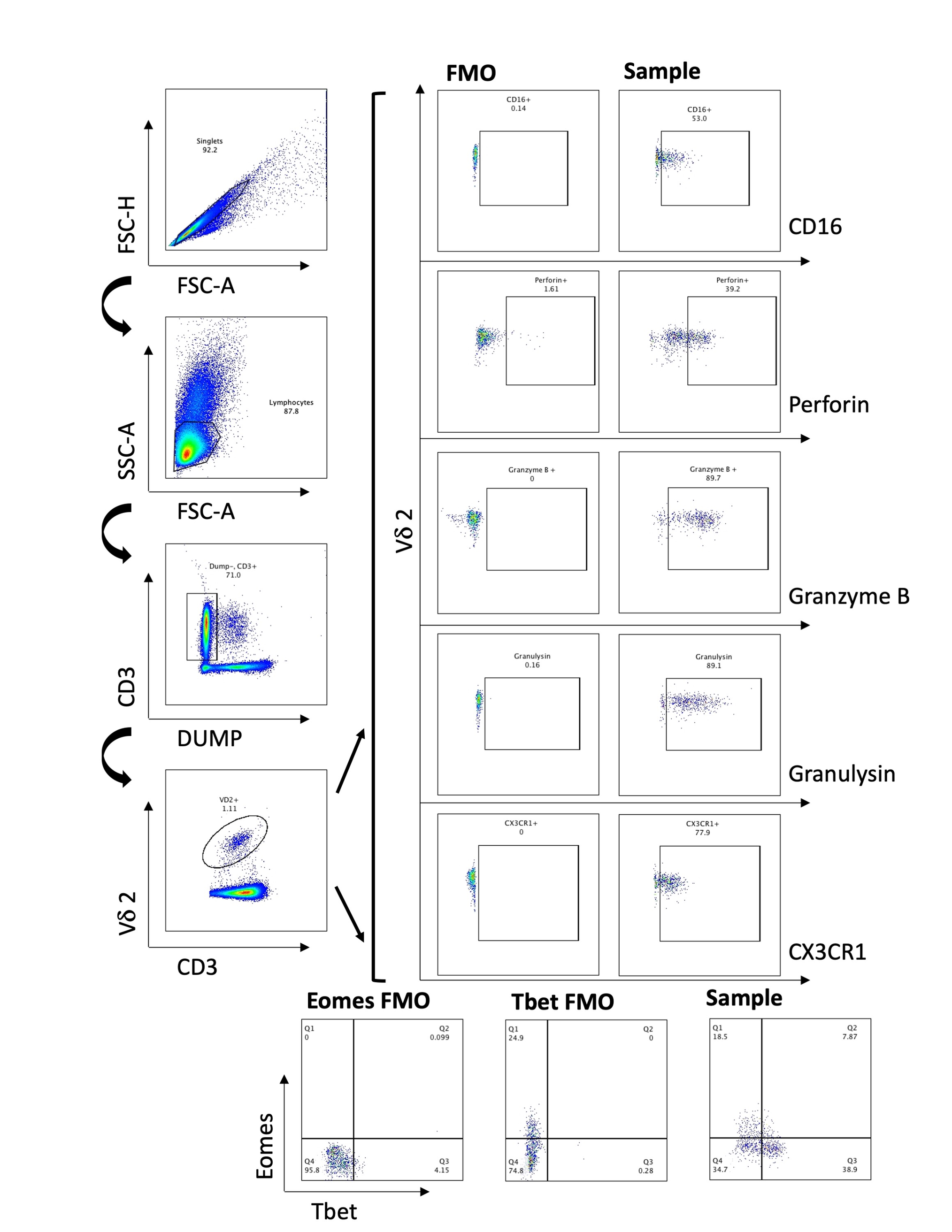

Supplement: S1 Fig — Vδ2 T cells evaluated for phenotypic markers of cytotoxicity were gated first by comparing FSC area to FSC height to exclude doublets, then by using FSC and SSC to select lymphocytes, then by excluding DUMP (CD14, CD19, Aqua live/dead) positive cells, and finally by selecting for CD3 positive and Vδ2 positive events. Fluorescence minus one (FMO) controls were used to define the gates for CD16+, GranzymeB+, Perforin+, Granulysin+, CX3CR1+, Eomes+, and Tbet+ events, as shown. (TIF) [file ppat.1008997.s002.tif]

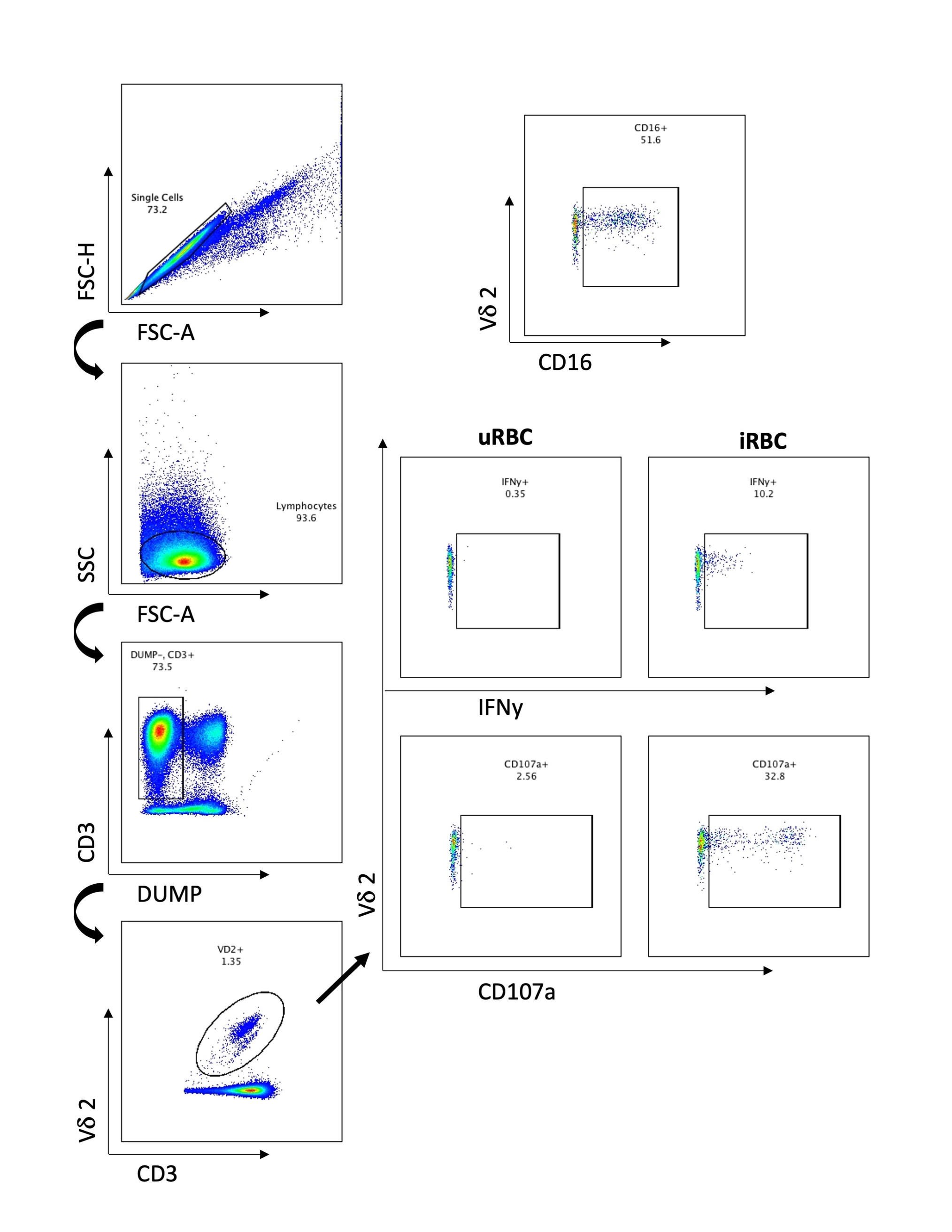

Supplement: S2 Fig — Vδ2 T cells evaluated for cytokine expression and degranulation after in vitro stimulation were gated first by comparing FSC area to FSC height to exclude doublets, then by using FSC and SSC to select lymphocytes, then by excluding DUMP (CD14, CD19, Aqua live/dead) positive cells, and finally by selecting for CD3 positive and Vδ2 positive events. uRBC or isotype controls were used to define IFNγ and CD107a positive events, as shown. (TIF) [file ppat.1008997.s003.tif]

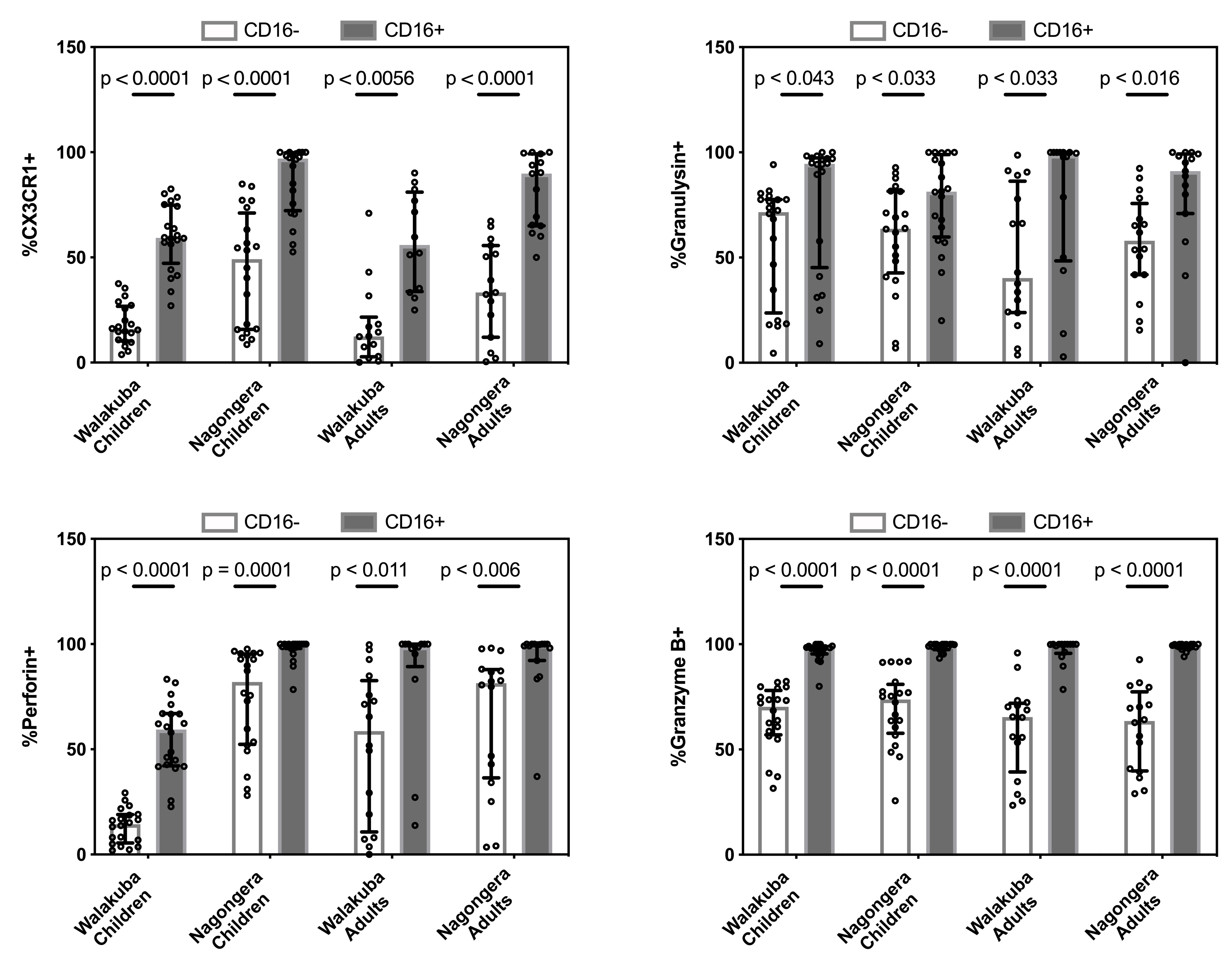

Supplement: S3 Fig — (A) The percentage of CD16+ and CD16-Vδ2 T cells expressing relevant cytotoxic markers, divided by age (children vs adults) and exposure history (high exposure = Nagogera, low exposure = Walakuba) (Walakuba children, n = 20; Nagongera children, n = 20; Walakuba adults, n = 13; Nagongera adults, n = 12). Scatter plots with median and IQR are shown. P values were determined by Wilcoxon matched pairs signed rank test. (TIF) [file ppat.1008997.s004.tif]

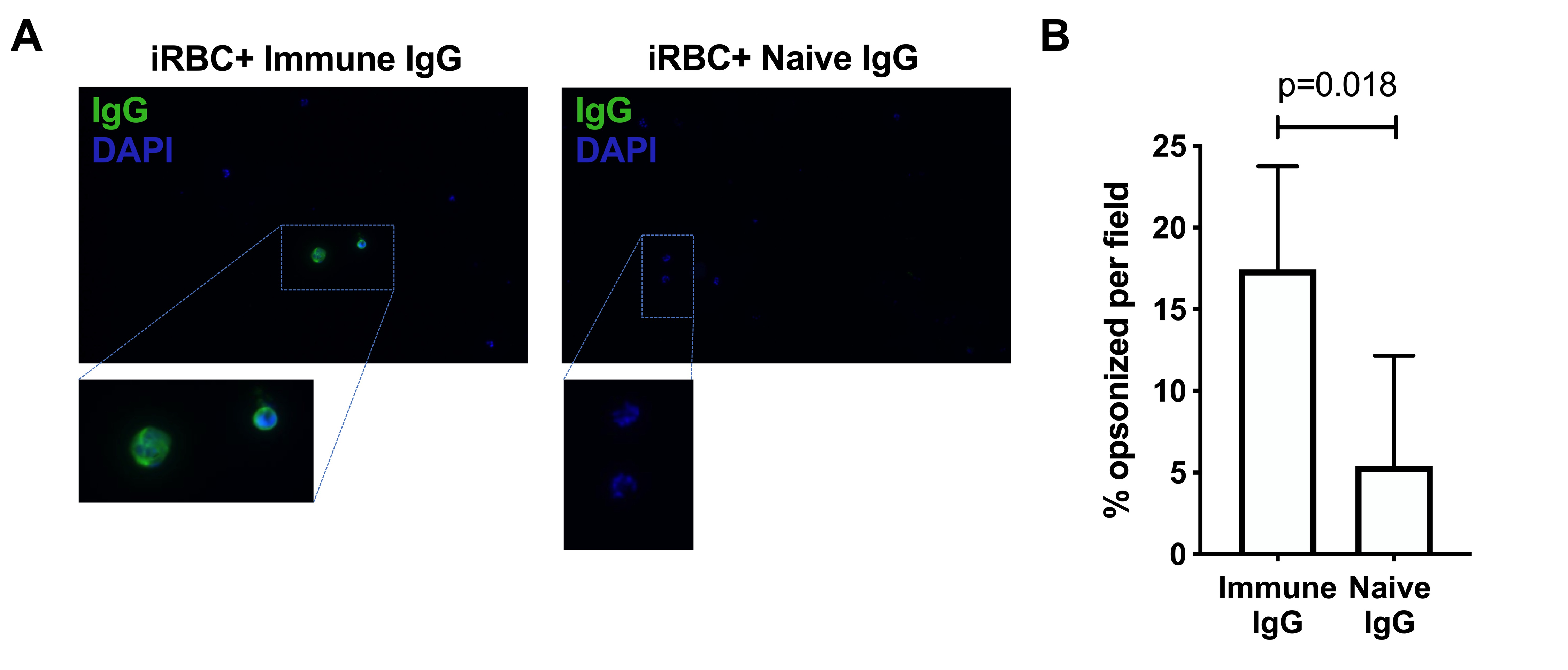

Supplement: S4 Fig — (A) Representative fluorescent images from the coincubation of iRBC with purified immune or naïve IgG (B) Quantification of the number of opsonized events per field (n = 20) in A. (TIF) [file ppat.1008997.s005.tif]

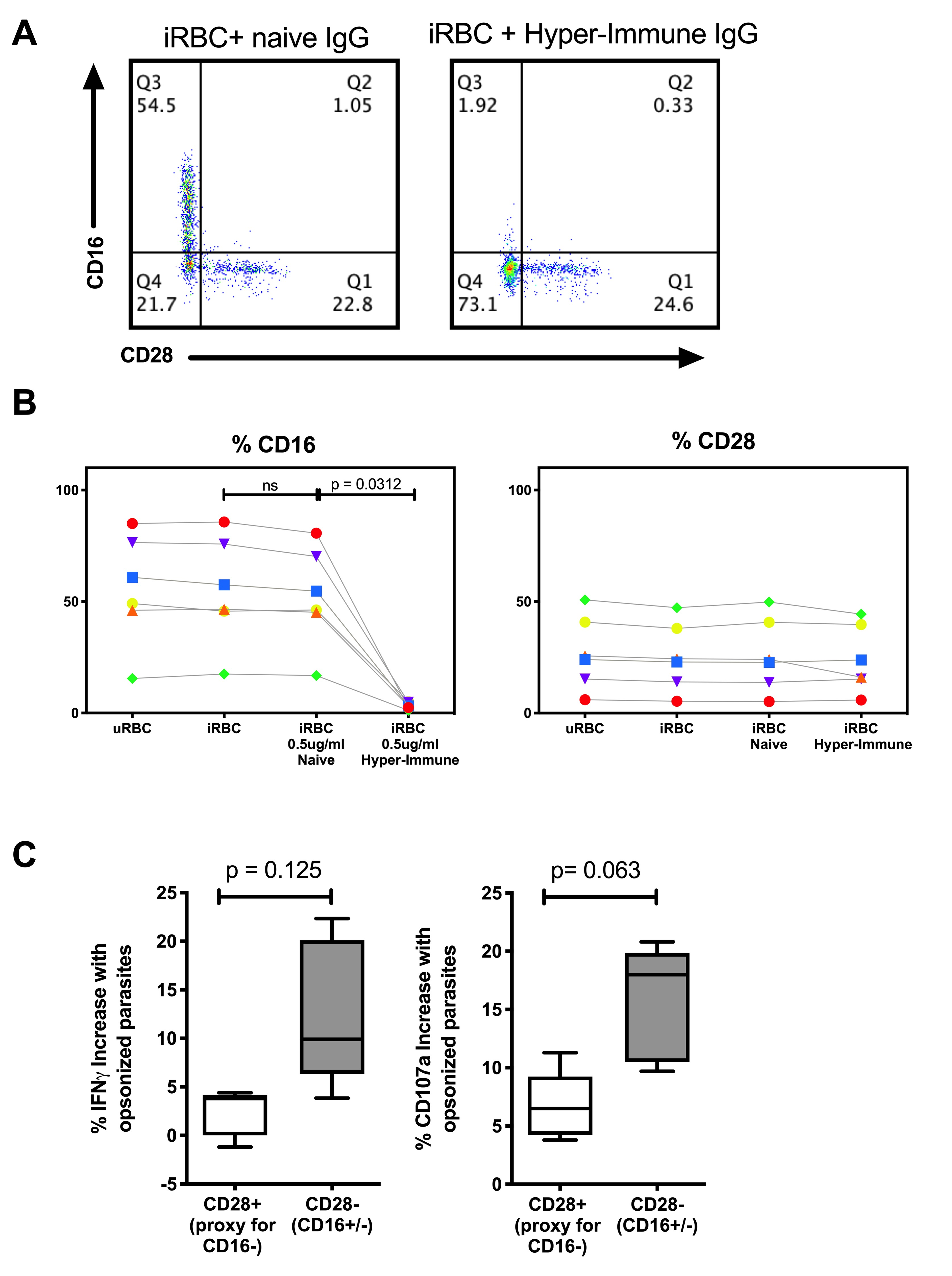

Supplement: S5 Fig — (A) Representative plot of CD16 vs CD28 expression on Vδ2 T cells after stimulation with iRBC+ naïve IgG or iRBC + hyper-immune IgG (B) Percent of Vδ2 T cells expressing CD16 or CD28 after the stimulation conditions listed. (C) The percent increase in IFNγ and CD107a+ Vδ2 T cells after stimulation with opsonized antigen (iRBC+ hyperimmune IgG) vs. unopsonized antigen (iRBC + naïve IgG), divided by CD28 expression (n = 6; p values determined by Wilcoxon matched pairs signed rank test). (TIF) [file ppat.1008997.s006.tif]
